# Object Specific Trajectory Optimization for Industrial X-ray Computed Tomography

**DOI:** 10.1038/srep19135

**Published:** 2016-01-28

**Authors:** Andreas Fischer, Tobias Lasser, Michael Schrapp, Jürgen Stephan, Peter B. Noël

**Affiliations:** 1 Siemens AG, Corporate Technology, 81730 Munich, Germany; 2 Computer Aided Medical Procedures (CAMP), Technische Universität München, 85748 Garching, Germany; 3 Department of Radiology, Technische Universität München, 81675 Munich, Germany; 4 Chair for Biomedical Physics and Institute for Medical Engineering, Technische Universität München, 85748 Garching, Germany

## Abstract

In industrial settings, X-ray computed tomography scans are a common tool for inspection of objects. Often the object can not be imaged using standard circular or helical trajectories because of constraints in space or time. Compared to medical applications the variance in size and materials is much larger. Adapting the acquisition trajectory to the object is beneficial and sometimes inevitable. There are currently no sophisticated methods for this adoption. Typically the operator places the object according to his best knowledge. We propose a detectability index based optimization algorithm which determines the scan trajectory on the basis of a CAD-model of the object. The detectability index is computed solely from simulated projections for multiple user defined features. By adapting the features the algorithm is adapted to different imaging tasks. Performance of simulated and measured data was qualitatively and quantitatively assessed.The results illustrate that our algorithm not only allows more accurate detection of features, but also delivers images with high overall quality in comparison to standard trajectory reconstructions. This work enables to reduce the number of projections and in consequence scan time by introducing an optimization algorithm to compose an object specific trajectory.

X-ray Computed Tomography (CT) is a widely used tool for inspection in industrial settings, in particular for nondestructive testing (NDT). It is used, for example, for dimensional metrology and defect detection. Apart from the non-existing radiation exposure issue, CT in NDT has several differences when compared to medical CT, such as large variations in object-size and attenuation. One common major issue is the importance of reduced scan times. This facilitates the expansion of industrial CT application from the laboratory to the factory with a full test coverage in the production line.

Currently, industrial CT almost exclusively uses standard circular or helical trajectories in combination with filtered backprojection (FBP) reconstruction algorithms. The increased availability of high performance computing hardware, for example GPUs^1^,^2^, facilitates the revival of iterative reconstruction algorithms, which inherently support arbitrary trajectories. To exploit the flexibility of iterative reconstruction methods, it makes sense to move towards trajectories that include “valuable” acquisition poses (position and orientation of the source/detector arrangement, often also called projections) and exclude “less valuable” acquisition poses. In Varga *et al.*^3^ it was demonstrated that not all acquisition poses have the same value for the reconstructed image. For example, for reconstruction of an edge at least one X-ray has to be tangential to the edge^4^. Furthermore, image quality can be severely reduced by artifacts due to beam-hardening and high attenuation materials. Typically it is not possible to avoid such artifacts completely, but one can choose a trajectory in such a way that the region of interest is not (or less) affected by the artifacts.

In a wide variety of industrial CT applications, prior knowledge of the investigated object is available, such as CAD-models of the object. This additional information can be utilized to determine a set of valuable acquisition poses for reconstruction from a limited amount of such poses in order to save time. In this paper we introduce an observer model based optimization algorithm with the goal to reduce the number of acquisition poses to only valuable poses for the reliable reconstruction of specific, user-defined features.

## Related Work

In recent years approaches inspired by compressed sensing^5^ significantly improved the reconstruction of objects from a sparse number of acquisition poses^6^,^7^,^8^,^9^,^10^. In contrast to those approaches, we focus on the optimization of the trajectory of acquisition poses before the actual data acquisition.

Some previous approaches adapt the trajectory dynamically during the acquisition process without having any explicit prior knowledge of the object. Placidi *et al.*^11^ propose an adaptive acquisition algorithm for Magnetic Resonance Imaging (MRI) that initially acquires images at 0, 45, 90 and 135 degrees. The entropy of the acquisition poses is then calculated and the pose between the two poses with the highest entropy difference are consecutively acquired. Batenburg *et al.*^12^ and Debravolski *et al.*^13^ use “information gain” to dynamically select valuable acquisition poses. They defined information gain as a measure on how much an additional acquisition pose reduces the range of possible solutions. Haque *et al.*^14^ adapt the angular step size during image acquisition. Inspired by the level crossing sampling scheme, they increase the sampling density at angular regions that contribute to the spectral richness or the information in the image. For intra-operative Single Photon Emission Computed Tomography (SPECT) Vogel *et al.*^15^ propose a greedy optimization method to incrementally add poses to the trajectory during acquisition, based on improving the numerical spectrum of the system matrix.

A different possibility is to use prior knowledge of the object to determine the trajectory before data acquisition. Verne *et al.*^16^ use a genetic algorithm to optimize a fitness function that assumes an elliptical object. It accumulates views that are along the main axis of the ellipse. This approach is only useful in a small range of applications as it is limited to elliptic shapes. Varga *et al.*^17^ optimize over a set of possible acquisition poses using the relative mean error between the real image and the reconstructed image. For optimisation of the fitness function a greedy and a simulated annealing algorithm are employed. A tangential ray is necessary to properly reconstruct an edge; thus, Zheng and Mueller^18^ assume prior knowledge of the object and base their approach on finding a minimal set of acquisition poses that cover tangential rays of the most relevant edges in the prior volume. Stayman and Siewerdsen^19^ use the information from a pre-operative high quality medical CT scan to optimize the trajectory for an intra-operative lower quality scan. They simulate the projection images from the pre-operative scan. With a greedy algorithm that selects the best acquisition pose in each iteration with respect to a local non pre-whitening observer model in combination with a frequency template, the trajectory is computed. For medical applications, they demonstrated the performance of a task-based trajectory versus standard trajectories to illustrate the advantage of integrating prior knowledge as well as the imaging task into customized acquisitions.

We extend the approach by Stayman *et al.*^19^ to industrial CT applications. In industrial CT we typically benefit from very accurate CAD data of the object to be reconstructed. This allows for trajectory optimization prior to acquisition without the need of a previous scan. Further we extend the approach from one point of interest to multiple user-defined points of interest. Although the local restriction to one point of interest is sufficient in many industrial CT applications, for example in weld joint investigations, there are applications (for example length measurements) where at least two points of interest are vital. In this paper we extend existing approaches by using CAD data in combination with several points of interest for optimization of an object specific trajectory with a low number of valuable acquisition poses.

## Method

The greedy trajectory optimization algorithm proposed in this paper finds an object specific optimal subset of acquisition poses out of a set of possible acquisition poses for optimal reconstruction of user defined features. We first introduce the notations for iterative reconstruction and detectability index, before presenting the trajectory optimization algorithm itself.

### Penalized Likelihood Reconstruction

The goal of CT reconstruction is to reconstruct the image 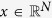 (a volume represented by a linearized vector) from its measurements 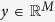. A common reconstruction method is Penalized Likelihood (PL) reconstruction.


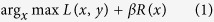


*L*(*x*, *y*) is the log-likelihood of volume *x* assuming measurements *y*. *R*(*x*) is a roughness penalty, which is weighted by *β* > 0. We use a quadratic space invariant penalty 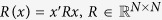
20. In the optimization process *x* is updated to maximize the likelihood that volume *x* has caused the particular measurement *y*. We use following log-likelihood model^21^:


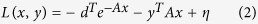


where each element *d*_*i*_ in the vector 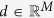 represents the numbers of photons leaving the tube in the direction of the a particular pixel. 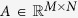 denotes the system matrix, which describes the relation between image space and the measurement space, while 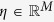 represents the noise due to scatter, detector noise etc. *M* is the number of measurement elements (number of detector elements × number of acquisition poses) and *N* is the number of image elements (voxels). The system matrix *A* implicitly contains all the geometry information, that is the acquisition poses or the trajectory.


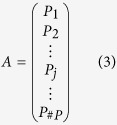


where #*P* denotes the number of acquisition poses and each matrix *P*_*j*_ corresponds to one acquisition pose. This notation emphasises that the trajectory (*A*) is a series of acquisition poses (*P*_*j*_). The goal of our optimization algorithm is to optimize the acquisition trajectory by composing it from valuable projections. Computed tomography reconstruction is typically ill posed^22^ and *A* is a huge matrix (on the order of several terabytes). This is why the optimization (1) is very challenging. For our work, we reconstruct the image *x* from the measurement data *y* using the convex PL algorithm with the quadratic penalty *R*^21^.

The trajectory optimization method we propose composes *A* from a set of acquisition poses 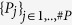 in order to keep it limited in size. Furthermore, the acquisition poses *P*_*j*_ should be valuable (like tangential views) and should avoid poses with limited information (like views with photon starvation). To facilitate this we employ a detectability index, which is described in the following.

### Detectability Index

We use a local non pre-whitening observer model as detectability index^19^. It is a measure on how good a signal provided by a frequency template can be discriminated from noise in a penalized likelihood CT reconstruction. In this paper the detectability index serves as a fitness measure for the trajectory optimization algorithm, in order to select the next best acquisition pose. To compute the detectability index, the Modulation Transfer Function (MTF) and the Noise Power Spectrum (NPS) are needed, which can be estimated from the raw measurements *y*^19^,^23^,^24^,^25^,


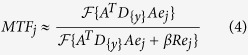



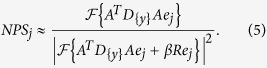


Here 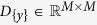 denotes a diagonal matrix with diagonal entries 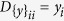 and 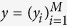. For *j* = 1, …, *N*, 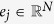 is the *j–th* unit vector, 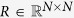 is a quadratic regularization matrix^26^, and *F* denotes the Fourier transform in three dimensions.

In both formulas, the acquisition poses enter through the system matrix *A*, and the feature dependency enters through the measurements in the diagonal matrix 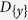. Using these formulas, we compute the detectability index^27^,^28^


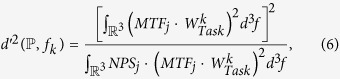


where 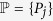 denotes the current set of acquisition poses as encoded in the system matrix *A*, and 
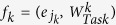
 describes the user-defined feature in terms of location 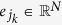 and structure 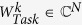 (a frequency template).

The frequency template 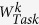 should match the Fourier transform of the expected signal *S*_*k*_ of the feature^27^, 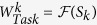. This way the frequencies in the *MTF* that match the frequency template *W* get a higher weight, and sets of acquisition poses that lead to a *MTF* with similar frequencies as the feature are more likely to be selected. The expected signal *S*_*k*_ is for example an edge in a specific direction. If there is no assumption about the signal structure, 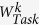 can be omitted or set uniformly to 1.

The local non pre-whitening observer model was picked for several reasons. It correlates well with human observer performance^27^. Edge directions can be incorporated by using the frequency template. This is especially valuable in industrial CT as there are usually distinct and previously known edges. It is very convenient as it can be computed only by its projections (simulated or measured). So there is no need to do complex system modelling.

### Trajectory Optimization Algorithm

To create the object specific trajectory, first a set of features 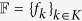 with 
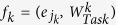
 has to be defined by the user. Then, the acquisition poses 
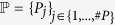
 for the object specific trajectory are computed by a greedy algorithm (see Algorithm 1), which uses the detectability index 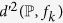 as a fitness measure.

**Figure 1:**
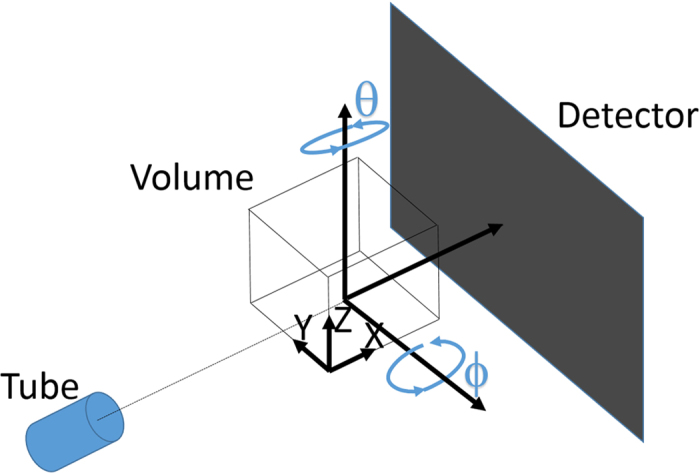
Schematic of the system geometry. The angles *θ* and *ϕ* describe the rotation of the object to be measured. One acquisition pose *P*_*j*_ is thus defined by an angle pair 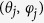.

As the algorithm is optimizing the trajectory before actual measurements, the measurements *y* needed to compute the detectability index (via the MTF/NPS estimates) have to be simulated from the CAD data for all possible acquisition poses 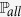. To limit the computational effort, we restricted the acquisition poses of the simulated measurements to only vary in the two rotation angles *θ* and *ϕ*, as illustrated in Fig. 1.

In every iteration of the algorithm, we then select for every feature *f*_*k*_ the acquisition pose 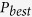 corresponding to the highest detectability index. Each of the selected acquisition poses changes the system matrix *A*, and therefore requires re-computation of the detectability index for every feature *f*_*k*_ in every iteration. The optimization process is stopped after a user-defined number of iterations.

To make sure that for each feature *f*_*k*_ with edge preference the view with rays tangent to this edge is covered, in the first iteration every feature *f*_*k*_ is processed individually. As the detectability index favors acquisition poses covering frequencies neglected by the previously selected acquisition poses, after the first iteration, all previously selected acquisition poses are taken into account for computation of the detectability index.


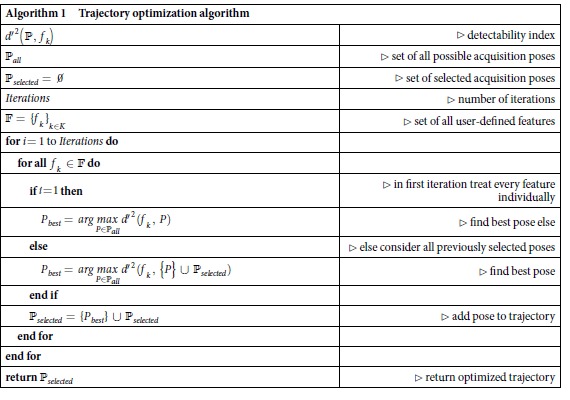


## Evaluations and Results

### Imaging Tasks

**Figure 2:**
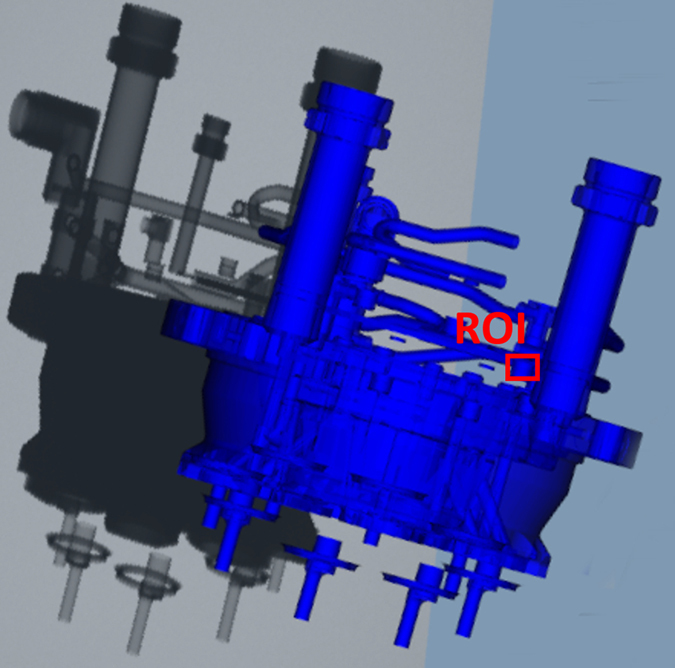
CAD models of the studied objects. The region of interest (ROI) of the steel object is marked in red. Both renderings are in false colors.

We applied the proposed trajectory optimization algorithm to two imaging tasks with different objects. The first task is the examination of a welded tube in a steel object, see Fig. 2a for a CAD dataset of the steel object. The second task is the measurement of the six faces of an aluminium cube with multiple drilled holes, see Fig. 2b for a CAD dataset of the aluminium cube.

The challenge for the steel object is to find a trajectory which does not suffer from photon starvation, as the region of interest (the welded tube) is very close to a massive steel plate, which is non-transparent for the employed 290 kVp X-ray tube voltage. For the measurement task with the aluminium cube there are six faces, necessitating the use of multiple features. As the density of aluminium is relatively low, photon starvation is not an issue. However, in order to reconstruct the faces correctly the angles of the acquisition poses are of high importance.

### Experimental Procedures

For both tasks and objects, the trajectory optimization algorithm is executed. For initialization of the algorithm, the measurements *y* for all possible acquisition poses 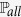 have to be simulated from the CAD data. These simulations were performed using the CAD data including material properties and aRTist^29^, a tool to quantitatively model X-ray imaging. The result of the trajectory optimization algorithm is a set of acquisition poses 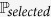.

For the steel object, measurements were simulated (again with aRTist) using 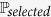 with 18 acquisition poses. For comparison, measurements were also simulated for traditional, uniform circle trajectories with 18 and 180 acquisition poses.

For the aluminum cube, measurements using 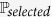 were both simulated (using 6, 12 and 18 acquisition poses) as well as measured on a real X-ray setup (using 6 acquisition poses). For comparison, real measurements and simulated measurements were also performed with the same number of acquisition poses on a traditional circle trajectory. In order to apply the optimized trajectory 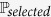 to the real measurement setup, the object in the scanner has to be registered with the CAD model. For this work, we did this by manually placing the aluminium cube in the scanner according to the position used for simulation.

All measurements were subsequently reconstructed using convex PL reconstruction^21^ with 40 iterations. Additionally, reference reconstructions with 100 iterations were performed from simulated data using 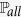 acquisition poses for both objects. For the reference reconstruction of the steel object we additionally removed the opaque components of the CAD model outside the region of interest, in order to prevent photon starvation. The reference reconstruction for the real measurements of the aluminum cube is an extensive scan with 1620 acquisition poses. The roughness penalty factor *β* was set to 0.4 in the case of the aluminum cube and 1.0 in the case of the steel object. Using these reference reconstructions as ‘ground truth’, the sum of squared differences (SSD) was computed for all other reconstructions.

### Results Steel Object

The only feature to be considered here is a welded joint at a tube, see Fig. 2a. Thus we have 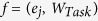, where *e*_*j*_ is chosen to match the region of interest (ROI), which is achieved by using a coarse 64^3^ resolution such that the ROI fits into one voxel *j*. The frequency template 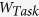 was chosen to be uniform, as defects of welded joints do not have particular structures.

For simulation of the measurements, a cone beam setting with a 290 kVp tube was used, with X-ray scattering based on the Monte Carlo method and a flat panel detector with 512^2^ pixels and a pixel size of 1.3mm. The source to object distance was 1400 mm, the source to detector distance 1950mm, while the angles (*θ*, *ϕ*) defining the acquisition poses were chosen as 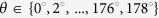 and 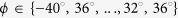, for a total of 3600 acquisition poses. The detectability index was computed using *β* = 1.0 on the 64^3^ volume.

**Figure 3:**
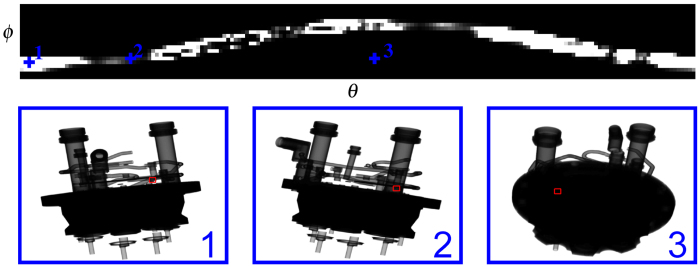
Map of detectability index. *Top:* the detectability index is drawn for all angle pairs (*θ*, *ϕ*), with white denoting a high value, and black a low value. *Bottom:* example simulated X-ray images are shown for three selected angle pairs: very good detectability with no overlaps (**1**), detectability affected by the metal tube (**2**), and overlaps of the ROI with the massive steel plate yielding very bad detectability (**3**).

Figure 3 shows the map of the detectability index for all angle pairs (*θ*, *ϕ*), along with simulated X-ray images at three selected angle pairs. It is obvious that photon starvation due to the massive steel plate is the main issue, and that the detectability index captures this very well.

**Figure 4:**
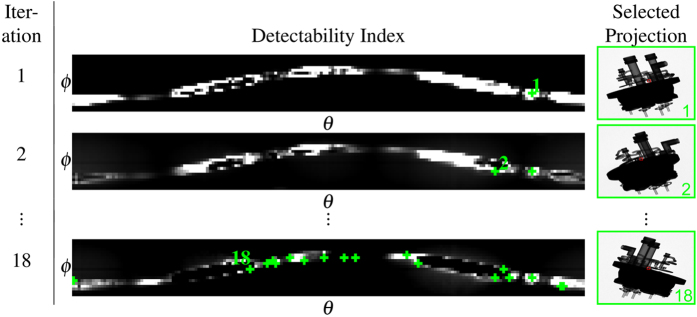
Illustration of the trajectory optimization algorithm. In each iteration the acquisition pose with the highest detectability index is selected. The right column shows the simulated measurement corresponding to the selected acquisition pose. The last row shows the final trajectory, consisting of 18 acquisition poses.

Figure 4 illustrates the execution of the trajectory optimization algorithm. In each iteration, the detectability map is computed for all angle pairs (*θ*, *ϕ*) based on the current system matrix *A*, and the acquisition pose with the highest index is added to the trajectory, updating the system matrix. For this experiment, the algorithm was stopped after 18 iterations, as we decided that this is a good trade-off between achievable image quality and acquisition time savings.

**Figure 5:**
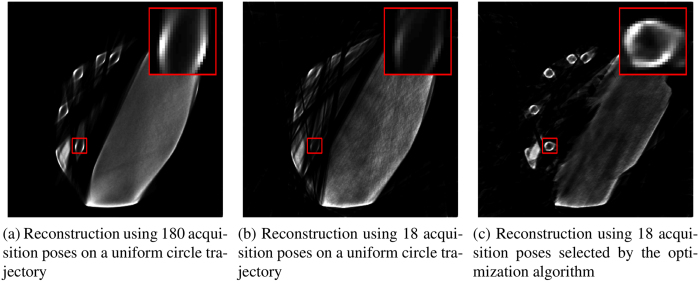
Reconstruction results of the steel object. Reconstructions using the traditional circle trajectory (**a**,**b**) suffer from severe translucency problems. With the optimized trajectory the circular tube is reconstructed properly (**c**).

Using the optimized trajectory 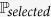 with 18 acquisition poses and simulated measurements, reconstructions were computed using a 412^3^ volume with 0.93 mm voxel size. Additionally, reconstructions were computed with the same settings, but using traditional, uniform circle trajectories using 18 and 180 acquisition poses. The results are shown in Fig. 5 with the ROI marked in red.

For the traditional circle trajectories it is not possible to properly reconstruct the ROI. This is due to the massive steel plate causing photon starvation. Using the optimized trajectory using 18 valuable acquisition poses, it is possible to completely recover the tube in the ROI. Table 1 shows the SSD between the simulated reference reconstruction and the experimental reconstruction results.

### Results Aluminium Cube

For the aluminium cube, the features 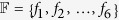 are the six faces of the cube. Each feature 
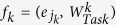
 is defined by its location *j*_*k*_ and by a frequency template 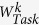 that prefers edges perpendicular to that face.

For simulation of the measurements, the parameters were chosen to match the capabilities of the real X-ray setup, which consisted of a Viscom XT9160-TED X-ray tube and a Perkin Elmer flat panel detector with 2048^2^ pixels and a pixel size of 0.2 mm binned to 512^2^ pixels. A cone beam geometry was used with a tube voltage of 150 kVp. The source to object distance was 12 0mm, the source to detector distance 820mm. The acquisition poses 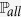 form a trajectory consisting of two single 360° circles, one around the Z-axis (Θ) and one around the Y-axis (Φ) with 540 acquisition poses each. The detectability index was computed with *β* = 0.4 on a 400^3^ volume with 0.117 mm voxel size. The trajectory optimization algorithm was executed with one, two or three iterations, yielding 6, 12 or 18 acquisition poses.

Using the optimized trajectory with 6, 12 or 18 acquisition poses and simulated measurements, reconstructions were computed using a volume tailored to the object with 380^3^ voxels and voxel size 0.117 mm. Additionally, measurements were simulated for traditional, uniform circle trajectories also using 6, 12 or 18 acquisition poses. All these reconstructions were compared against the simulated reference reconstruction using SSD, see Table 1.

**Figure 6:**
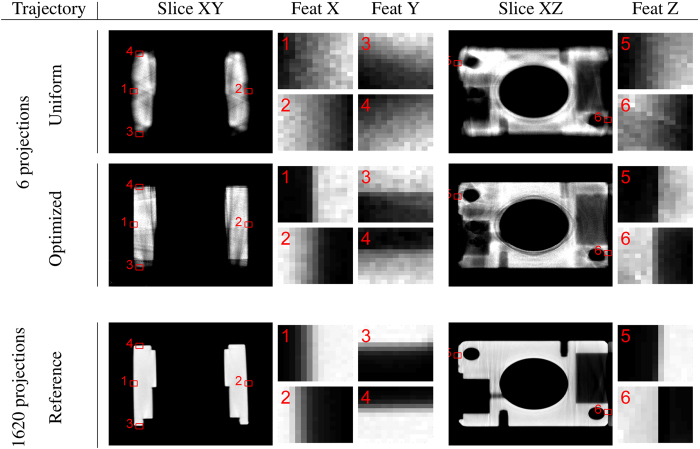
Results: Aluminium Cube Measured Data. Comparison of reconstruction result of the aluminium cube from uniform sampling on a circle, with the optimized trajectory and the reference reconstruction from 1620 measured projections. The six feature points with the edges in X, Y and Z directions are enlarged.

For the optimized trajectory and the traditional circle trajectory, each with 6 acquisition poses, measurements were also performed at the real X-ray setup. The resulting reconstructions (using the same settings as above) are shown in Fig. 6. For reference, a reconstruction of measured data with 1620 acquisition poses is also shown. Table 1 shows the SSD between the experimental reconstructions and the reference reconstructions for both the simulated measurements and the real measurements.

The reconstructions from the optimized trajectory perform better than reconstructions from the circle trajectory, see Fig. 6. Even with only six acquisition poses, our proposed method provides superior results with much sharper edges, because the optimization algorithm selects acquisition poses with rays tangential to the planes of the cube. Quantitatively the proposed method also performs better as indicated by Table 1. The results for the multiple iterations of the optimization algorithm (12 and 18 acquisition poses) also show that the proposed algorithm works well for multiple features and that there is no significant negative interaction between the features.

## Discussion

In this paper we introduced an observer model based trajectory optimization algorithm for determining optimal, object-specific trajectories with a low number of valuable acquisition poses for industrial CT. Our solution takes into consideration a CAD model of the object to be imaged as well as multiple user defined areas of interest (features). Compared to traditional trajectories (circular or helical) coupled with filtered backprojection reconstructions, we can automatically choose a sparse set of valuable acquisition poses and provide a significantly improved image quality. This allows us to significantly reduce the scan time, while also reducing a wide range of image artifacts. The trajectory optimization algorithm itself has a runtime on the order of several hours as implemented by us on a regular desktop computer (optimizations are of course still possible). But in cases of repetitive scans like inline CT applications, or in cases like the steel object, where the results from the optimized trajectory are superior to a circular scan with a high sampling rate, this effort is worthwhile.

A practical drawback of our study is that a registration is necessary between the CAD model and the object placed in the X-ray scanner. This issue can be solved simply by acquiring an initial X-ray image and performing a 2D/3D registration between the initial X-ray image and the CAD model. However, this was not within the scope of this work and will be addressed in future studies. In future work one could also invest more time in the optimization and use different optimization algorithms like leave one out and redesign. This may further improve the optimized trajectory.

The requirements for industrial CT are significantly different than for medical CT applications. Major challenges in medical CT with regard to issues like radiation exposure^30^ are not valid for industrial CT. However, time is a critical issue for both CT applications, and is often more challenging in industrial CT because of the size and density of objects. The most prominent feature of industrial CT is the availability of additional and reliable knowledge about the object to be imaged. In medical CT it is very challenging to perform a reliable and robust registration between pre-operative data and a current acquisition. In this work we have leveraged all available information (CAD model, user defined features) to generate fast and reliable 3D reconstructions.

The proposed algorithms delivers quantitative and qualitative good results for objects with specific features of interest. To reconstruct a complex object as a whole, the possible advantages of an optimized trajectory over a standard trajectory with uniform sampling shrink. However, in industrial CT applications often only distinct regions of the object need to be reconstructed with good quality, like weld joints or critical parts on circuit boards.

Industrial CT has great potential and offers many advantages, especially in delivering higher quality images and more information in comparison to current inspection techniques. Because of the advance of acquisition and reconstruction speeds, the employment of industrial X-ray tomography systems as a standard inspection tool will increase over the next decades. We believe the availability of an algorithm like the one proposed in this work will facilitate these investigations, thus expanding the number of potential users. In conclusion, our observer model based trajectory optimization algorithm is a promising new algorithm that provides many advantages in terms of image quality and imaging speed.

## Additional Information

**How to cite this article**: Fischer, A. *et al.* Object Specific Trajectory Optimization for Industrial X-ray Computed Tomography. *Sci. Rep.* **6**, 19135; doi: 10.1038/srep19135 (2016).

## Figures and Tables

**Table 1:** Quantitive comparison of optimized and circle trajectories.

Steel Object (single feature)								
**Trajectory**	**Data**	**Feature**						
Uni18	Sim	8.425						
Uni180	Sim	5.914						
Opt18	Sim	3.726						
**Aluminium Cube (multiple features)**								
Trajectory	Data	Feat 1	Feat 2	Feat 3	Feat 4	Feat 5	Feat 6	Sum
Uni6	Sim	0.596	0.423	0.732	2.766	0.514	0.751	5.783
Uni12	Sim	0.445	0.364	0.671	0.650	0.361	0.547	3.038
Uni18	Sim	0.373	0.396	0.382	0.056	0.338	0.193	1.738
Opt6	Sim	0.134	0.114	0.475	0.190	0.133	0.096	1.142
Opt12	Sim	0.105	0.138	0.243	0.123	0.137	0.104	0.849
Opt18	Sim	0.070	0.098	0.275	0.116	0.077	0.097	0.733
Uni6	Real	0.413	0.246	0.697	0.540	0.277	0.923	3.096
Opt6	Real	0.104	0.107	0.332	0.414	0.128	0.184	1.269

Sum of squared differences (SSD) of a 13^3^ region around the features compared to the reference reconstructions.
